# Effect of Phthalates and Their Substitutes on the Physiology of *Pseudomonas aeruginosa*

**DOI:** 10.3390/microorganisms10091788

**Published:** 2022-09-05

**Authors:** Mélissande Louis, Ali Tahrioui, Julien Verdon, Audrey David, Sophie Rodrigues, Magalie Barreau, Maëliss Manac’h, Audrey Thiroux, Baptiste Luton, Charly Dupont, Marie Le Calvé, Alexis Bazire, Alexandre Crépin, Maximilien Clabaut, Emilie Portier, Laure Taupin, Florian Defontaine, Thomas Clamens, Emeline Bouffartigues, Pierre Cornelis, Marc Feuilloley, Jocelyne Caillon, Alain Dufour, Jean-Marc Berjeaud, Olivier Lesouhaitier, Sylvie Chevalier

**Affiliations:** 1 Unité de Recherche Communication Bactérienne et Stratégies Anti-Infectieuses, CBSA UR4312, Université de Rouen-Normandie, Normandie Université, F-27000 Évreux, France; 2 SéSAD, Fédération de Recherche “Sécurité Sanitaire, Bien Être, Aliment Durable”, Université de Rouen-Normandie, Normandie Université, F-27000 Évreux, France; 3 IMPERIAL Project Consortium, ANSES, F-94706 Maisons-Alfort, France; 4 CNRS UMR7267 Ecologie et Biologie des Interactions (EBI), Université de Poitiers, F-86000 Poitiers, France; 5 Université de Bretagne-Sud, EA 3884, LBCM, IUEM, F-56100 Lorient, France; 6 EA3826 Thérapeutiques Cliniques et Expérimentales des Infections, Faculté de Médecine, Université de Nantes, F-44000 Nantes, France

**Keywords:** phthalates, *Pseudomonas aeruginosa*, virulence, biofilm, antibiotic susceptibility

## Abstract

Phthalates are used in a variety of applications—for example, as plasticizers in polyvinylchloride products to improve their flexibility—and can be easily released into the environment. In addition to being major persistent organic environmental pollutants, some phthalates are responsible for the carcinogenicity, teratogenicity, and endocrine disruption that are notably affecting steroidogenesis in mammals. Numerous studies have thus focused on deciphering their effects on mammals and eukaryotic cells. While multicellular organisms such as humans are known to display various microbiota, including all of the microorganisms that may be commensal, symbiotic, or pathogenic, few studies have aimed at investigating the relationships between phthalates and bacteria, notably regarding their effects on opportunistic pathogens and the severity of the associated pathologies. Herein, the effects of phthalates and their substitutes were investigated on the human pathogen, *Pseudomonas aeruginosa*, in terms of physiology, virulence, susceptibility to antibiotics, and ability to form biofilms. We show in particular that most of these compounds increased biofilm formation, while some of them enhanced the bacterial membrane fluidity and altered the bacterial morphology.

## 1. Introduction

*Pseudomonas aeruginosa* is an opportunistic pathogen that is responsible for a wide range of acute and chronic infections of lungs, especially in cystic fibrosis patients, urinary tract, burn wounds, and in immunocompromised patients [1]. This bacterium is intrinsically resistant to a broad range of antibiotics and there has been an increase in the frequency of multidrug resistant strains, leading to the classification of *P. aeruginosa* among the six major human pathogens (ESKAPE), and the three most critical bacterial species [2]. *P. aeruginosa* can be found in different environments associated with human activity, such as in soils or water [3], or with eukaryotes such as nematodes, plants, and mammals [4], mainly because of its versatile metabolism. The latter is related to its large genome (6.3 Mbp), encoding numerous complex regulatory networks and allowing *P. aeruginosa* to perceive and to adapt to several physicochemical signals such as variations in temperature, osmotic pressure, and surfaces of various natures (PVC, glass, etc.), as well as to eukaryotic signals such as hormones, or effectors of the immune system [5].

*P. aeruginosa* is able to switch from free-living (planktonic) to sessile (biofilm) lifestyles and vice versa, depending on the environmental signals perceived—these lifestyles being associated with acute and chronic infections, respectively [5]. Free-living *P. aeruginosa* can produce numerous secreted virulence factors, including the blue-pigmented pyocyanin, promoting the colonization and the dissemination of the bacterium, and the fluorescent siderophore pyoverdine that is required for providing environmental Fe^3+^ to the cell [6]. Many of these virulence factors are tightly regulated by the three interconnected quorum-sensing (QS) communication systems, two of which (the Las and Rhl systems) are based on the production and sensing of *N*-acyl-homoserine lactones (AHL), and the PQS system, which depends on 4-hydroxy-2-alkylquinolines (HAQ) [7]. Chronic infections are mostly associated with biofilms, which are organized communities of microorganisms bound to a surface or interface and/or to each other and are embedded into a self-produced exopolymeric matrix consisting of exopolysaccharides, extracellular DNA, outer membrane vesicles, and proteins [8,9], in association with secretions from infected tissue [10]. *P. aeruginosa*, similar to most bacteria, is able to form biofilms at the air/liquid interface known as pellicles [11]. This lifestyle is commonly associated with stress conditions, in which the bacteria protect themselves from the immune system components and from xenobiotics such as antibiotics.

Phthalates, or phthalic acid esters, are stable lipophilic plasticizers used in the formulation of polyvinylchlorides (PVC) to improve their flexibility [12]. These compounds are largely used in numerous manufactures, including car industries (soft plastics), cosmetics (shower gels, shampoo, and perfumes), food containers, and medical devices (catheters, blood bags, and gastro-resistant capsules). Because they are not covalently linked to the PVC matrix, they can be easily released into the environment and are thus major environmental pollutants. Human activities and urbanization have increased the discharge of phthalic esters into atmospheric and aquatic environments, and the use of agricultural plastics has exacerbated soil contamination in rural areas [13]. They can be found in soils at the concentration of 0.03–1280 mg·kg^−1^, in sediments at 0.0003–218 mg·kg^−1^, in drinking water at 0.16–170 μg·L^−1^, in atmospheric air at 0.4–65 ng·L^−1^, in indoor air at 20–240 ng·L^−1^, in wastewater at 0.0004–58.3 g·L^−1^, and in dust at 2.38–4.1 g·kg^−1^ [14,15,16,17]. As PVC is part of common food containers, phthalates are among the migrating molecules frequently found in food, and ingested doses can reach 18 μg·kg^−1^·day^−1^ [18]. In hospitals, the measured exposure doses were estimated to reach 0.14 mg·kg^−1^·day^−1^ for adults and 2.5 mg·kg^−1^·day^−1^ for newborns [18].

In addition to being major persistent organic environmental pollutants, some phthalic esters are responsible for the carcinogenicity, teratogenicity, and endocrine disruption notably affecting steroidogenesis in mammals [19]. They indeed display structural homologies with some hormones [20] and are referred as xenoestrogens or endocrine disruptors, noticeably because they can bind to nuclear receptors such as estrogen receptor alpha (ERα), beta (ERβ), peroxisome proliferator–activator receptor gamma (PPARγ), and aryl hydrocarbon receptor (AhR), thereby interfering with the endocrine response [20]. Besides this effect, an increasing number of studies tend to show that phthalates could also promote infections. For example, prenatal exposure to bis-(2-ethylhexyl)-phthalate (DEHP) increases the risk of pneumopathologies in young children [21]. Chronic exposure to DEHP dysregulates normal B-lymphoid development in salmonids, may increase susceptibility to infection [22,23], and exacerbates inflammatory occurrence in the lungs [24].

The relationship between infections and phthalates may be indirect, as the consequence of their effect on the immune system modulation, causing the suppression [25] or improvement [26] of macrophage activities, or modifying the patterns of cytokine production [27,28,29]. In addition to these detrimental effects on mammalian endocrine and immune systems, phthalates could also exert a direct effect on the bacterial physiology and/or virulence. Although this topic has not yet attracted a lot of attention, the following data suggest that it deserves deeper investigations. Indeed, dibutyl phthalate (DBP) and DEHP were shown to inhibit the ability of the phytopathogen *Pectobacterium carotovorum* ssp. *carotovorum* to form biofilms [30], suggesting that DBP can be perceived by bacteria, leading to adaptation to this compound. In line with this hypothesis, DEHP was shown to increase *Helicobacter pylori* cytotoxicity [31]. Dimethyl phthalate (DMP) was shown to affect *Pseudomonas fluorescens* biological functions in terms of growth and energy metabolism, and to alter the bacterial membrane [32]. Noticeably, bacteria, including *P. aeruginosa*, are able to sense human hormones, a property at the origin of the concept of microbial endocrinology [33,34,35,36], suggesting that bacteria would also be able to perceive molecules that are structurally related to hormones. For instance, *P. aeruginosa* exposure to human hormones such as the C-type natriuretic peptide [37], norepinephrine [38], and epinephrine [39] resulted in increased production of virulence factors [37,38,39]. *Lactobacillus crispatus* strains V4 and CIP104459 were shown to respond to estradiol by increasing biosurfactant production and adhesion to vaginal VK2/E6E7 cells [40]. In addition, strain V4 increased its aggregation abilities, while strain CIP104459 increased its membrane fluidity in response to estradiol exposure [40]. Noticeably, an estrogen-binding protein was previously identified in *P. aeruginosa* [41], and estradiol at human physiological concentrations can affect *P. aeruginosa* mucoidy [42], a phenotype resulting from the overproduction of the exopolysaccharide alginate, which plays a major role in the establishment of chronic infections in the lungs of cystic fibrosis patients [43,44].

The above cited publications support the notion that phthalates can affect pathogenic bacteria, which might in turn modify their infectious properties. This study thus aims to thoroughly investigate the effects of a variety of phthalates and of molecules used as phthalate substitutes on *P. aeruginosa* physiology and virulence-related traits.

## 2. Materials and Methods

### 2.1. Reagents

The reagent-grade compounds (Sigma-Aldrich, St. Louis, MI, USA) were solubilized in ethanol (100%) to prepare a 1 M working solution. In most experiments performed in this study, the molecules were used at concentrations ranging from 10^−10^ to 10^−3^ M in 0.1% ethanol (final concentration).

### 2.2. P. aeruginosa Strains, Growth Medium, and Monitoring of P. aeruginosa Growth

*Pseudomonas aeruginosa* H103, a prototroph of the PAO1 wild-type strain [45], was grown in M9 succinate medium (Na_2_HPO_4_ 6 g·L^−1^; KH_2_HPO_4_ 3 g·L^−1^; NH_4_Cl 1 g·L^−1^; NaCl 0.5 g·L^−1^ in distilled H_2_O), supplemented with 1 mM of MgSO_4_ containing 20 mM of succinate as carbon source. The control condition consisted in M9 succinate medium, supplemented with ethanol at 0.1% in each assay. An overnight culture of *P. aeruginosa* H103 (18 h) was grown in M9 succinate medium at 37 °C with shaking (180 rpm) and adjusted to an A_580_ value of 0.08. Then, the untreated (control ethanol 0.1%) and treated (with the molecules of interest at various concentrations) H103 cell suspensions were grown in M9 succinate medium for 24 h at 37 °C with shaking (180 rpm). The A_580_ was monitored in 96-well microtiter plates every 30 min using the Spark 20 M multimode Microplate Reader controlled by Spark Control^TM^ software Version 2.1 (Tecan Group Ltd., Crailsheim, Germany). The data were plotted, and each point indicates the mean ± standard deviation (SD) of A_580_ values.

### 2.3. Determination of Minimum Concentration of Phthalates Inhibiting the Growth of P. aeruginosa Clinical Isolates

The phthalates and phthalate substitutes, at concentrations ranging from 10^−3^ to 10^−10^ M, were evaluated for their effects on the growth of *P. aeruginosa* clinical strains. Twenty-five clinical strains of *P. aeruginosa* isolated from lower respiratory tract sites of different cystic fibrosis-suffering patients were collected. The minimal concentrations of several phthalates (DEHP, DEHT, DBP, DEP, and TXIB) inhibiting the growth of these clinical strains, and *P. aeruginosa* ATCC 27853 as reference strain, were determined by the microdilution technique using Mueller Hinton broth (MHB) containing 0.1% final concentration of ethanol [46]. Overnight MHB cultures of *P. aeruginosa* clinical isolates were used to prepare inocula of 10^5^ CFU·mL^−1^. The tests were performed in duplicate. The minimal concentration was defined as the lowest concentration of a phthalate preventing turbidity after 24 h incubation at 37 °C.

### 2.4. Antibiotic Susceptibility Assays

Minimal inhibitory concentration (MIC) is the lowest concentration of the antibiotic that prevents visible bacterial growth. MICs of tobramycin and ceftazidime were assessed as previously described [46], using antibiotic concentrations ranging from 0.25 to 16 µg·mL^−1^ in Mueller Hinton broth (MHB) containing 0.1% ethanol. To evaluate the effects of phthalates and substitutes on the susceptibility of *P. aeruginosa* to the two antibiotics, the assays were performed as a classical MIC experiment, except that phthalates or substitutes were added in each well at 10^−3^ M in MHB containing 0.1% ethanol (final concentration). The *P. aeruginosa* ATCC 27853 strain was used as a reference strain, and bacteria were added at a final concentration of 10^5^ CFU·mL^−1^. After 24 h incubation at 37 °C, MICs of ceftazidime and tobramycin, alone or supplemented by a phthalate, were read based on the EUCAST guidelines. The tests were performed in duplicate.

### 2.5. Virulence Factor Assays

#### 2.5.1. Pyocyanin

Pyocyanin was quantified from *P. aeruginosa* cultures upon treatment or not with phthalates or phthalate substitutes. Cultures were performed in King A medium (Sigma Aldrich^®^, Carlsbad, CA, USA) in microtiter plates or in M9 succinate in Erlenmeyer (5 mL culture in a 50 mL Erlenmeyer). The absorbance of the extracted pyocyanin from culture supernatants was measured at 520 nm and normalized to the bacterial growth (A_580_).

#### 2.5.2. Pyoverdine

Pyoverdine was quantified from *P. aeruginosa* cultures upon treatment or not with phthalates or phthalate substitutes. Cultures were performed in King B medium (Sigma Aldrich^®^) in microtiter plates. The control consisted of growth medium containing 0.1% ethanol (final concentration). The absorbance of the culture supernatants was measured at 405 nm and normalized to the bacterial growth (A_580_).

### 2.6. Biofilm Assays

#### 2.6.1. Polystyrene Microtiter Plate Assays

*P. aeruginosa* H103 biofilm cultures were grown on 24-well polystyrene microplates (Thermo Fisher Scientific, Nunc^TM^, Waltham, MA, USA). Aliquots of 1 mL of bacterial suspension from an overnight culture, which were washed twice with a physiological sterile solution (NaCl 0.9%) and adjusted to an A_580_ value of 0.1, were placed into each well, avoiding the peripheral wells of the microplate. Then, the microplate was incubated for 2 h at 37 °C without shaking to allow the attachment of the bacterial cells onto the polystyrene surface. After incubation, the physiological sterile solution containing unbound cells was carefully removed and 1 mL of M9 succinate medium containing 10^−3^ M of the phthalates or phthalate substitutes in 0.1% ethanol was added. Biofilm cultures of *P. aeruginosa* H103 without the studied compounds (M9 succinate medium supplemented with ethanol at 0.1%) were used as a control condition. The biofilm cultures were incubated for 22 h at 37 °C without shaking. After incubation, cell growth was determined by A_580_. Biofilm amount was measured by discarding the medium, rinsing the wells with water, and staining any bound cells with crystal violet at 0.1% for 15 min. The dye was dissolved in 30% *v*/*v* acetic acid and A_595_ was recorded using the Spark 20 M multimode microplate reader controlled by Spark Control^TM^ software Version 2.1 (Tecan Group Ltd., Crailsheim, Germany). In each experiment, background staining was adjusted by subtracting the crystal violet bound to uninoculated control wells.

#### 2.6.2. Pellicle Formation

Overnight cultures were diluted in M9 succinate to an A_580_ of 0.08. A volume of 3 mL of this bacterial suspension, either containing or not phthalates or phthalate substitutes at 10^−3^ M, was added into borosilicate glass tubes and incubated at 37 °C for 24 h without shaking. Pellicles were observed at the air–liquid interface of the culture.

#### 2.6.3. Glass-Bottom Plate Assays and Biofilm Observation with Confocal Laser Scanning Microscopy (CLSM)

Overnight planktonic cultures were diluted to an A_580_ value of 1 and placed on glass-bottom microplates. *P. aeruginosa* biofilm cultures were grown for 24 h at 37 °C in static conditions in M9 succinate medium, either supplemented or not with the phthalates or phthalate substitutes. Prior to image acquisition, bacteria were labeled adding 5 µM of SYTO^®^ 9 green-fluorescent nucleic acid stain (Invitrogen, Thermo Fisher Scientific, Waltham, MA, USA; excitation at 488 nm and emission from 500 to 550 nm) prepared in sterile physiological solution (0.9% NaCl), incubated at room temperature for 15 min in the dark, and washed twice with fresh medium. The CLSM observations were carried out with a Zeiss LSM710 (Carl Zeiss Microscopy, Jena, Germany), using a 40-× oil immersion objective. Images were taken every micrometer throughout the whole biofilm depth. For visualization and processing of three-dimensional (3D) images, the ZEN 2.1 SP1 ZEN software (https://www.zeiss.com/microscopy/int/downloads/zen.html; accessed on 18 July 2022) (Carl Zeiss Microscopy) was used. Quantitative analyses of image stacks were performed using the COMSTAT software (http://www.imageanalysis.dk/; accessed on 18 July 2022) [47]. At least three image stacks from at least three independent experiments were used for each analysis.

### 2.7. Scanning Electron Microscope Analyses

The morphology of *P. aeruginosa* was studied by scanning electron microscopy using a TENEO Volume Scope microscope (FEI, Hillsboro, OR, USA) under 10 kV. Briefly, *P. aeruginosa* was grown at 37 °C for 24 h in M9 succinate medium under agitation (180 rpm), in Erlenmeyer (5:50, *v*/*v*, medium/Erlenmeyer volume), in the absence (control) or presence of the phthalates or phthalate substitutes at 10^−3^ M (or 0.1% ethanol for the control). Each sample was then collected by centrifugation (7500× *g*, 10 min), and the fixation was performed by immersing the bacterial pellets for 1 h in 1 mL 2.5% glutaraldehyde in 1 M phosphate buffer (pH 7.1). Sample preparation was then carried out as previously described [48].

### 2.8. Membrane Fluidity Measurement by Fluorescence Anisotropy

Membrane fluidity was determined by measuring fluorescence anisotropy of *P. aeruginosa* H103 [49] with or without 10^−3^ M of phthalates or phthalate substitutes. Planktonic cultures grown for 24 h at 37 °C with shaking (180 rpm) were centrifuged at 7500× *g* for 10 min at room temperature (RT) and washed twice with a MgSO_4_•7H_2_O solution at 10 mM. Then, one µL of the 1,6-diphenyl-1,3,5-hexatriene probe (DPH, Sigma Aldrich^®^), at 4 mM was added to the bacterial suspensions adjusted to an A_580_ value of 0.1 and incubated for 30 min in the dark at 37 °C. After incubation, measurement of fluorescence polarization was performed using the Spark 20 M multimode microplate reader equipped with a polarizer controlled by the Spark Control^TM^ software Version 2.1 (Tecan Group Ltd.) (excitation wavelength: 365 nm; emission wavelength: 425 nm). The anisotropy (*r*) was calculated with the following equation: *r* = (*I*1 − G × *I*2)/(*I*1 + 2 × G × *I*2), where (*I*1) corresponds to the emission fluorescence intensity measured alongside and (*I*2) to the emission fluorescence intensity measured perpendicularly to the light excitation plan. G corresponds to the G factor. The relationship between anisotropy and membrane fluidity is an inverse one, wherein decreasing anisotropy values correspond to a more fluid membrane and vice versa.

### 2.9. Statistical Analysis

The data were statistically analyzed using ordinary one-way ANOVA followed by Dunnett’s multiple comparison test (anisotropy assays) or two-samples paired two-sided *t*-test to calculate *p* values with GraphPad Prism (GraphPad Prism 9 version 9.4.0; GraphPad Software, San Diego, CA, USA). All values were reported and plotted as means with SD or SEM of at least triplicate analyses for each experimental variable. NS = Not Significant; *p* > 0.05; * *p* < 0.05; ** *p* < 0.01; *** *p* < 0.001; **** *p* < 0.0001.

## 3. Results

### 3.1. Phthalates and Substitutes

**Figure 1:**
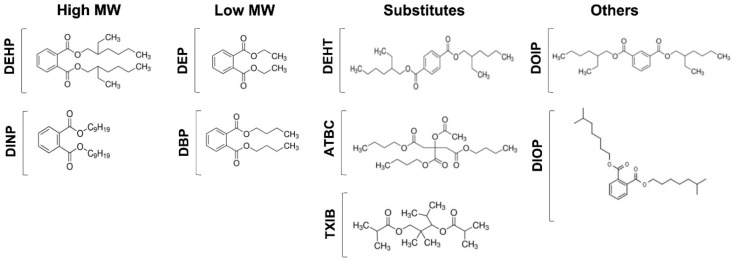
**Phthalates and substitutes used in this study.** High molecular weight phthalates: DEHP (2-éthylhexyl)-phthalate), DINP (bis (7-methyloctyl) phthalate). Low molecular weight phthalates: DEP (diethyl phthalate), and DBP (dibutyl phthalate). Substitutes: DEHT (bis (2-ethylhexyl) terephthalate), ATBC (tributyl acetylcitrate), and TXIB (2,2,4-trimethyl-1,3-pentanediol diisobutyrate). Other substitutes: DOIP (di-2-ethylhexyl isophthalate) and DIOP (dicyclohexylphthalate).

In this study, phthalates, and phthalate substitutes (Figure 1), in concentrations ranging from 10^−3^ to 10^−10^ M, were evaluated for their effects on *P. aeruginosa* physiology. Phthalates are phthalic acids esters, which are classified into two groups based on their molecular weight. Phthalates with low molecular weight (LMW) and short alkyl chains of one to five carbons, such as diethyl phthalate (DEP) and dibutyl phthalate (DBP), are often used as solvents in personal care products, cosmetics, and pharmaceuticals, and as plasticizers in non-PVC products, including textiles, paints, solvents, adhesives, and food packaging. Phthalates with high molecular weight (HMW) display longer alkyl chains of five or more carbons. This class includes the bis-(2-ethylhexyl)-phthalate (DEHP) and the diisononyl phthalate (DINP or bis (7-methyloctyl) phthalate). They are mainly used as plasticizers in PVC products, including in medical devices and children’s toys, and in construction materials, clothing, furniture, rubber, glues, or mastics [50,51]. Since some of these phthalates (including DEHP, DBP, DINP), have been classified as carcinogenic, mutagenic, and reprotoxic, phthalate substitutes that are considered less toxic, with limited volatility and release of materials, have been developed. This class includes the bis (2-ethylhexyl) terephthalate (DEHTP or DEHT), acetyl tributyl citrate (ATBC), and the 2,2,4-trimethyl-1,3-pentanediol diisobutyrate (TXIB). In addition to these molecules, the bis-2-ethylhexyl isophthalate (DOIP) and the bis-isooctylphthalate (DIOP, bis-cyclohexylphthalate), for which there is not enough information to date regarding a potential effect as endocrine disruptors, were also part of the panel of molecules tested (Figure 1).

### 3.2. Exposure to Phthalates and Substitutes Did Not Affect P. aeruginosa Growth Kinetic

**Figure 2:**
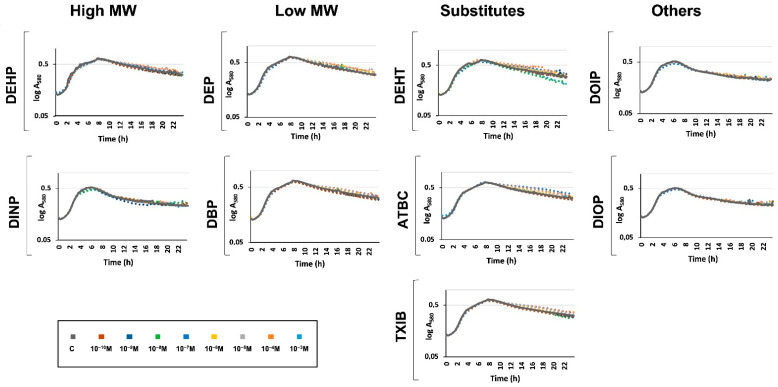
**Phthalates or substitutes did not affect *P. aeruginosa* growth**. Compounds were added at concentrations ranging from 10^−10^ to 10^−3^ M to the growth medium prior to *P. aeruginosa* inoculation. Growth was monitored for 24 h. GC: growth control in M9 containing succinate as sole carbon source without any phthalates or substitutes.

Since the aim of this study was to evaluate the physiological response to phthalates and their substitutes on *P. aeruginosa* H103, we first attempted to grow *P. aeruginosa* H103 in M9 minimal medium containing 10^−3^ M of each of the compounds as a sole carbon source, this concentration being the highest tested in the subsequent experiments. No bacterial growth could be observed (data not shown), indicating that *P. aeruginosa* H103 was not able to metabolize these compounds in these conditions, or not enough at this concentration to enable bacterial growth. *P. aeruginosa* was then grown in M9 succinate medium supplemented with either phthalates or substitutes at a concentration ranging from 10^−3^ to 10^−10^ M. In this environment, the carbon source used by *P. aeruginosa* can only be succinate [49,52]. By this way, phthalates or substitutes should not be used as carbon sources, and their effects should rather reflect a physiological adaptation of *P. aeruginosa* upon exposure. The control condition was conducted in M9 succinate medium supplemented with ethanol at a final concentration of 0.1%. As shown in Figure 2, it appears that adding phthalates or substitutes did not affect *P. aeruginosa* growth in M9 succinate medium at all the concentrations tested, indicating that they were neither used as carbon sources nor impaired *P. aeruginosa* H103 growth in our conditions.

### 3.3. Exposure to Phthalates and Substitutes Did Not Affect Minimal Inhibitory Concentrations of Antibiotics towards P. aeruginosa

Next, we investigated the effects of phthalates and substitutes on the minimal inhibitory concentrations of two antibiotics that are commonly used to treat *P. aeruginosa*-related infections. To this aim, the growth of twenty-five clinical strains isolated from lower respiratory tract sites of different cystic fibrosis patients was assayed in MHB medium containing ethanol at 0.1% final concentration by the microdilution technique [53], in the presence of DEHP, DEHT, DBP, DEP, or TXIB, at concentrations ranging from 10^−3^ to 10^−10^ M. These experiments were performed in MHB since this medium is commonly used to determine MICs (EUCAST, https://www.eucast.org; accessed on 18 July 2022) such as *P. aeruginosa* H103 (Figure 2). No growth alteration was observed for the clinical strains in our conditions (data not shown). Ceftazidime (CAZ) and tobramycin (TM) MICs were then determined in the presence of DEHP, DEHT, DBP, DEP, or TXIB at a final concentration of 10^−3^ M. As shown in Table 1, MICs of CAZ and TM were not altered in the presence of these five compounds for most of the strains. Of note, while not significant, we observed a tendency for a slightly decreased susceptibility of strain P40 towards CAZ upon exposure to DEHT, DEP, TXIB, or DBP, suggesting that, in some cases, phthalates and some substitutes may increase resistance to some antibiotics.

### 3.4. Phthalates and Substitutes Did Not Deeply Alter the Production by P. aeruginosa of Two Major Virulence Factors

**Figure 3:**
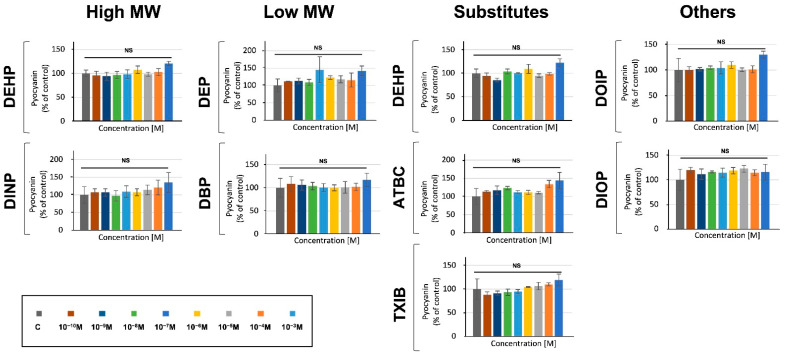
**Phthalates or substitutes did not alter pyocyanin production**. Phthalate and substitute concentrations ranging from 10^−3^ to 10^−10^ M (colors indicated in the scale). GC: *P. aeruginosa*’s growth control in M9 succinate medium without phthalates or substitutes. NS = *p* > 0.05.

We further investigated the effects of phthalates and substitutes on *P. aeruginosa* virulence by measuring the production of two major virulence factors, i.e., pyocyanin and pyoverdine. To this aim, *P. aeruginosa* exposure to phthalates or substitutes was performed at concentrations ranging from 10^−3^ to 10^−10^ M and pyocyanin production was measured after 24 h of growth in microtiter plates, as described in the materials and methods section. In a first attempt, we assayed its production in M9 succinate medium, and neither *P. aeruginosa* exposure to phthalates, nor to substitutes, led to pyocyanin production alterations (data not shown). To ascertain these data, an additional assay was performed in King A medium to promote the production of pyocyanin [54]. In this condition, no drastic effect was detected at any of the concentrations of the molecules tested (Figure 3). Although we noted a tendency of the studied molecules to slightly increase pyocyanin production in a dose-dependent manner, this effect remained not significant in our conditions (Figure 3).

**Figure 4:**
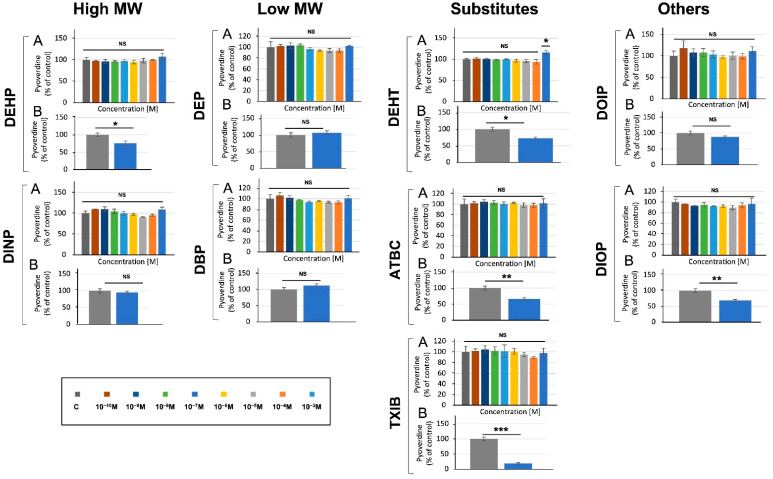
Effect of phthalates and substitutes on pyoverdine production when *P. aeruginosa* was grown in microtiter plates (**A**) or in Erlenmeyer flasks (**B**). Phthalate and substitute concentrations ranging from 10^−3^ to 10^−10^ M (colors indicated in the scale). GC: *P. aeruginosa*’s growth control inM9 succinate medium without any phthalates or substitutes. NS = *p* > 0.05; * = *p* < 0.05; ** = *p* < 0.01; *** = *p* < 0.001.

As in the case of pyocyanin, assays performed in M9 succinate medium did not lead to any alteration in pyoverdine production by *P. aeruginosa* (data not shown). Assays were then performed in King B medium to allow the detection of pyoverdine while lowering that of pyocyanin [54], and no effect was detected in response to *P. aeruginosa* exposure to the compounds (Figure 4A). Since pyoverdine production depends partly on oxygenation [6], we next assayed its production when *P. aeruginosa* was grown in M9 succinate medium in Erlenmeyer (5:50, *v*/*v*, medium/Erlenmeyer volume) upon exposure to 10^−3^ M phthalates or substitutes, or without treatment. In these conditions, aeration of the cultures was improved. Exposure to TXIB, ATBC, and DIOP—and, to a lower extent, to DEHP and DEHT, but not to the other compounds—significantly reduced pyoverdine production by 81, 40, 35, 27, and 23% (Figure 4B), suggesting an effect of these compounds on *P. aeruginosa* acute virulence abilities.

### 3.5. Phthalates and Substitutes Affect P. aeruginosa Biofilm Formation

**Figure 5:**
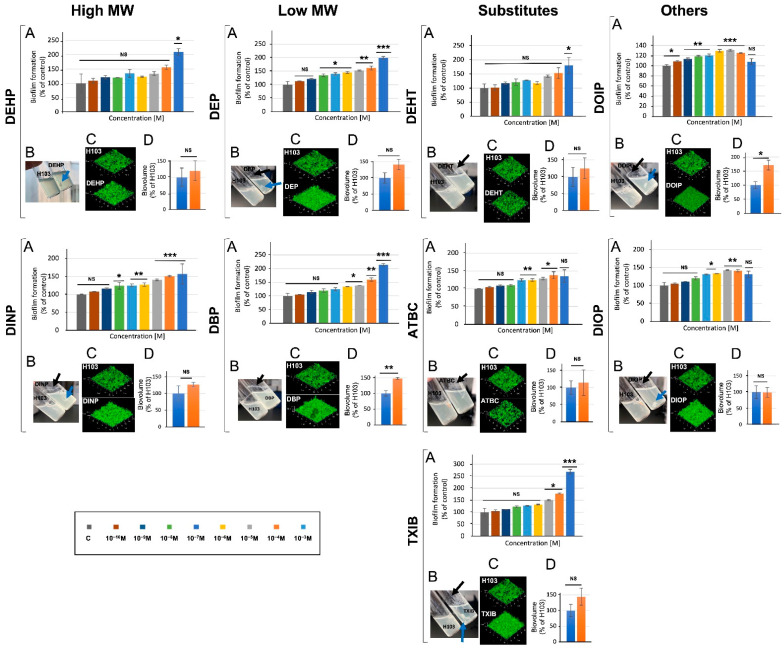
**Phthalates and substitutes affect biofilm formation**. Biofilms were investigated in polystyrene plates (**A**), at the air–liquid interface (**B**), by confocal laser scanning microscope observation (**C**), and by COMSTAT analysis of their biovolumes (**D**). NS = *p* > 0.05; * = *p* < 0.05; ** = *p* < 0.01; *** = *p* < 0.001.

To assess the effect of phthalates and their substitutes on the ability of *P. aeruginosa* to form biofilm, the bacteria were grown for 24 h in 24-well microplates in M9 succinate medium, supplemented or not with 10^−3^ M of the studied compounds (Figure 5A). Remarkably, each of the nine studied compounds caused a significant increase in biofilm formation, in a dose-dependent manner for most of them. The most important effect was observed for the TXIB substitute, which increased the biofilm formation 2.8-fold at 10^−3^ M; the low MW DBP and DEP and the high MW DEHP and DEHT all enhanced this ability about 2-fold. Accordingly, pellicle formation was enhanced upon exposure to each compound (Figure 5B, black arrows). In addition to increasing pellicle production, most compounds (i.e., DEHP, DINP, DEP, DEHT, TXIB, DOIP, and DIOP), also caused the formation of bacterial aggregates in liquid cultures (Figure 5B, blue arrows). CLSM observations of biofilms (Figure 5C) and COMSTAT image analysis of their biovolumes (Figure 5D) supported these data. However, the biofilm increase was significant only upon DBP and DOIP exposure. Altogether, these data indicate that the assayed phthalates and substitutes can promote biofilm growth.

### 3.6. Phthalate and Substitute Exposure Affect P. aeruginosa Membrane Fluidity

**Figure 6:**
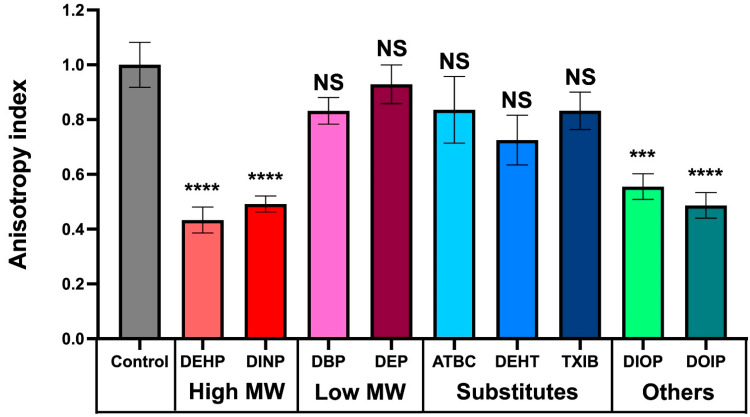
**Effect of phthalates and substitutes on membrane fluidity.** Anisotropy index variations were observed upon addition of phthalates or substitutes at 10^−3^ M to *P. aeruginosa*. Graphs represent means ± SEM. NS = *p* > 0.05; *** = *p* < 0.001, **** = *p* < 0.0001.

Because the phthalates and substitutes used in this study affect the physiology of *P. aeruginosa* in a similar way, i.e., by increasing to some extent the biofilm formation abilities in a dose-dependent manner, we suspected that they could act at least through a nonspecific mode of action. Since the compounds of interest are fat-soluble molecules, it is plausible that they interact with *P. aeruginosa* membranes. We therefore investigated *P. aeruginosa* membrane fluidity upon exposure to 10^−3^ M of each compound by fluorescence polarization assays using a 1,6-diphenyl-1,3,5-hexatriene (DPH) fluorescent probe, as previously described [49,55,56]. The relationship between anisotropy and membrane fluidity is an inverse one, where decreasing anisotropy values correspond to a more fluid membrane and vice versa. As depicted on Figure 6, the high MW phthalates, DEHP and DINP, and the other substitutes, DIOP and DOIP, caused an increase in the membrane fluidity of *P. aeruginosa*. However, the other tested compounds had only a limited and not significant effect.

### 3.7. Effect of Phthalates and Substitutes on P. aeruginosa Morphology

**Figure 7:**
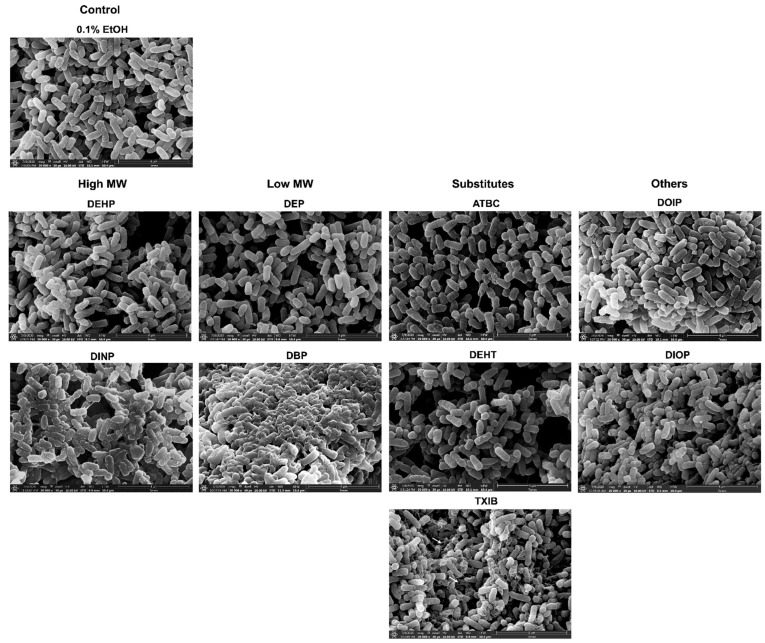
**Some phthalates can alter the morphology of *P. aeruginosa*.** Bacteria were observed by scanning electron microscopy after being grown for 24 h in M9 succinate medium under agitation in the presence of phthalates or substitutes at 10^−3^ M. The micrographs presented are representative of 10 images taken for each sample obtained from a biological duplicate. The white arrows indicate the presence of holes in the bacterial envelope. The magnification is ×20,000 and the bars represent 4 µm.

To gain insight into the effects of phthalates and substitutes on *P. aeruginosa* morphology, a scanning electron microscope approach was used. Representative electron micrographs of selected experiments are shown in Figure 7. Most of the phthalates tested (ATBC, DEHP, DEHT, DEP, DIOP, and DOIP) did not cause visible changes on the *P. aeruginosa* surface when compared to the solvent alone. Morphology alterations were mainly noticeable upon DBP, TXIB, and DINP treatment. The bacterial envelope appeared wrinkled, and abundant vesicle-like structures and ramifications between cells were observed upon DINP exposure, and some crystals were observed. In presence of TXIB, the bacterial envelope appeared roughened or invaginated, and some bacteria also showed apparent holes (Figure 7, TXIB, white arrows). Treatment with DBP led to an important modification of the bacterial morphology, since the cells appeared sticky, and probably embedded into a kind of exopolymeric matrix (Figure 7, DBP). Altogether, these data suggest that phthalates and substitutes affect the morphology of *P. aeruginosa*.

## 4. Discussion

Phthalates are major organic pollutants that result mainly from anthropic activity and are found in many environments. *P. aeruginosa* is an opportunistic pathogen that can be found in environments associated with human activity, such as soils or water [3,14], suggesting that the bacterium may be exposed to numerous pollutants such as phthalates. While numerous studies have focused on the effects of phthalates as endocrine disruptors on humans or animals, few have attached importance to their possible effects on bacterial physiology. Herein, the impacts of phthalates and their substitutes were investigated on the human opportunistic pathogen, *P. aeruginosa,* in terms of physiology and virulence-related phenotypes (production of virulence factors, biofilm formation ability, and susceptibility to antibiotics).

In this study, we have shown that the phthalates and their substitutes were not used as carbon sources by *P. aeruginosa*. Succinate being the preferred carbon source of *P. aeruginosa* [49,52], we chose a growth-minimal medium including succinate to avoid the metabolization of other possible carbon sources because of the catabolic repression that prevents the utilization of the less-preferred carbon sources for as long as succinate is not fully consumed [49,52]. Thus, the effects of the studied compounds may reflect a physiological adaptation of *P. aeruginosa* upon exposure. Several *Pseudomonas* species, which have been isolated from contaminated soils such as industrial mining areas or intensive crop lands [38], are indeed able to metabolize phthalates, including DBP [57,58], which is one of the most toxic phthalic acid esters [59]. This ability is of particular interest in terms of bioremediation and soil decontamination [60]. In the soil-isolated *Pseudomonas* sp. Strain, DNB-S1, two metabolic pathways, namely the protocatechuate pathway and the gentisate pathway, were identified to metabolize DBP [61]. In addition, we have shown that the phthalates and their substitutes did not affect the growth of *P. aeruginosa* H103, nor that of 25 clinical strains isolated from lower respiratory tract sites of different cystic fibrosis patients. However, some phthalates may influence the growth of bacteria in other conditions, since dimethyl-phthalate (DMP) at a concentration range of 20 to 40 mg·L^−1^ (corresponding to about 1 to 2 × 10^−4^ M) can inhibit the growth of *P. fluorescens* and glucose utilization [32].

The effects of the phthalates and their substitutes were then investigated with regards to phenotypes related to *P. aeruginosa* virulence. Pyocyanin and pyoverdine are among the major virulence factors that are produced and secreted by *P. aeruginosa*. Pyocyanin is a blue phenazine pigment that generates production and accumulation of reactive oxygen species causing oxidative stress to susceptible prokaryotic and eukaryotic cells [62]. This virulence factor enables *P. aeruginosa* maintenance and persistence over other microbes in environments [63], and provokes eukaryotic cell apoptosis and senescence [64,65]. We have shown that the phthalates and their substitutes used herein did not lead to any drastic effect on pyocyanin production, neither in M9 succinate minimal medium, nor in King A medium which promotes pyocyanin production [54]. Pyoverdine is a green, fluorescent, high-affinity siderophore that is required for chelating the essential nutrient Fe^3+^ from the bacterial environment prior to the uptake of the ferrisiderophore complex via a TonB-dependent specific receptor [66]. The production of pyoverdine is controlled at least partly by PvdS, an extracytoplasmic function sigma factor, which also controls the expression of the genes encoding two other virulence factors: exotoxin A and the PrpL-secreted protease [44,67,68]. Thus, pyoverdine production can be used as a marker of the ability to induce acute virulence. In M9 minimal medium, as well as in King B medium, which enhances pyoverdine production [54], no effect on pyoverdine production by *P. aeruginosa* was detected in response to exposure to the compounds. However, when enhancing aeration of the bacterial cultures, exposure to TXIB, ATBC, and DIOP—and to lower extents to DEHP and DEHT—significantly reduced pyoverdine production, suggesting an effect of these compounds on *P. aeruginosa* acute virulence abilities. Since pyoverdine is a high-affinity siderophore, these data also suggest that such treatments led to reduced Fe^3+^ availability by a mechanism that is still unknown. Interestingly, it was recently shown that the ethyl acetate fraction of culture supernatants of the marine sponge symbiont, *Brevibacterium casei*, reduced *P. aeruginosa* pyoverdine production by about 30% [69]. Noticeably, one of the major constituents of *Brevibacterium casei* supernatant was identified as DEP [69]. However, DEP had no effect on pyoverdine production in our study.

The effects of the phthalates and their substitutes were next investigated with regards to the ability of *P. aeruginosa* to form biofilm. Whatever the type of biofilm (biofilms on solid surfaces of polystyrene or glass; pellicle at the air–liquid interface) and the technique used to observe and/or quantify the biofilms (crystal violet assay or use of fluorescent stains prior to CLSM observation; visual observation of pellicles), each of the nine studied compounds caused a significant increase in biofilm formation. These data can be at least partly related to the SEM analysis. Indeed, we observed vesicle-like structures that are common components of biofilm matrixes [70,71], and/or ramifications between cells, which could be related to the matrixial exopolysaccharides [72]. In agreement with our data, some phthalates, including DMP, di-n-hexyl phthalate (DnHP), and DEHP, were previously shown to promote *P. aeruginosa* biofilm formation and resistance to the disinfectant chlorine [73]. Exposure to indoor dust including phthalate esters enhanced biofilm formation by almost two-fold in *Escherichia coli, Enterococcus faecalis*, and *P. aeruginosa,* without impacting the bacterial growth rates [74], which is in line with our data. In contrast, DBP and DEHP reduced biofilm formation by *Clavibacter michiganensis* ssp. *sepedonicus* and *Pectobacterium carotovorum* ssp. *carotovorum* [30]. Altogether, these data suggest that phthalates or substitutes may have an impact on biofilm formation.

We also noticed the presence of bacterial aggregates in liquid cultures upon exposure to DEHP, DINP, DEP, DEHT, TXIB, DOIP, and DIOP, which could originate from altered bacterial surface properties [75]. Membrane alterations, such as membrane wrinkling, were noticeable mainly upon DBP, TXIB, and DINP treatment. Membrane deformations were also reported for *E. coli* K12 [76] and *P. fluorescens* [32] upon DMP exposure. *Lactobacillus plantarum* displayed a smooth appearance upon DBP treatment [77], but no case of breakdown or lysis has ever been reported to our knowledge. Since phthalates are fat-soluble compounds, we suspected that they could interact with the bacterial membrane, which may alter the cell envelope integrity. Using assays based on fluorescence anisotropy, we have shown that, among the nine studied compounds, only DEHP, DINP, DIOP, and DOIP led to increased membrane fluidity in *P. aeruginosa*. Noticeably, membrane fluidity alterations were shown to impact attachment and biofilm formation [78]. Sex steroids have been found to induce membrane stress responses in *P. aeruginosa* [79], and in some strains of *Lactobacillus crispatus*, exposure to 17β-estradiol led to increased membrane fluidity and adhesion to vaginal mucosa cells [40]. Similarly, *P. aeruginosa* that was infected by the filamentous phage Pf4, displayed increased membrane fluidity and biofilm formation [55]. Altogether, these data indicate that cell envelope alterations may lead the bacteria to protect themselves by switching to a community-based lifestyle. However, it is noticeable that the main effect on biofilm formation was observed for DEP, DBP, TXIB, and, to a lesser extent, for ATBC and DEHT, for which the fluorescence anisotropy did not show a significant variation. Interestingly, the surface hydrophobicity and membrane permeability of *P. fluorescens* were increased by DMP [32]. One must thus assume that membrane alterations related to the compound structures may only partly explain their mode of action, and that further studies are required to acquire deeper insights into the molecular effect of these compounds on *P. aeruginosa* physiology.

Finally, we also have shown that the MICs of ceftazidime and tobramycin towards *P. aeruginosa* were not modified when bacteria were treated with DEHP, DEHT, DBP, DEP, or TXIB. However, *P. aeruginosa* strain P40 was slightly, but not significantly, more resistant to ceftazidime upon exposure to DEHT, DEP, TXIB, and DBP, suggesting that phthalates and some substitutes may, in some cases, increase resistance to some antibiotics. To the best of our knowledge, this is the first report of a putative relationship between phthalates and antibiotic susceptibility. However, considerably more studies will need to be conducted to determine the correlation between virulence mechanisms and antibiotic resistance in *P. aeruginosa* under exposure to environmental pollutants. The relationship between antibiotic resistance, biofilm formation, or virulence factor production is far from clear [80]. This is of particular interest since there are conflicting observations in the literature regarding a correlation between *P. aeruginosa* resistance to antibiotics and selected virulence traits. Indeed, on one hand, recent contradictory studies suggest that variations in the expression of virulence factors, particularly biofilm formation, are not correlated with antibiotic resistance in MDR *P. aeruginosa* strains [81] or, on the opposite, are statistically correlated with virulence traits in a panel of clinical isolates [82]. All these data reinforce the interest in studying potential correlations between xenobiotics and virulence traits, especially from a mechanistic point of view, in order to develop new control strategies to counter infections.

To conclude, the effects of phthalates and their substitutes were investigated in this study on the human pathogen *P. aeruginosa* in terms of physiology, virulence, susceptibility to antibiotics, morphology, membrane fluidity, and ability to form biofilms. We have shown that exposure to some phthalates or substitutes can modulate *P. aeruginosa* behavior, notably in terms of biofilm formation and, for some of them, also in terms of the siderophore pyoverdine production, morphology, or membrane fluidity alterations. The molecular mechanisms leading to these phenotypes upon phthalate or substitute exposure could be different from one compound to another and will require further investigations to be deciphered. However, since *P. aeruginosa* is able to sense human hormones such as the natriuretic peptides [37,83], dynorphin [38], serotonin [84], epinephrine [39], or estradiol [41,42], for example, and since phthalates are considered as xenoestrogens, it is possible that *P. aeruginosa* can sense and respond to such compounds by adjusting its behavior and physiology.

## Figures and Tables

**Table 1:** Effects of DEHP, DEHT, DEP, TXIB, and DBP (10^−3^ M) on *P. aeruginosa* clinical strain susceptibility towards ceftazidime (CAZ) and tobramycin (TM). MICs are given in µg·mL^−1^.

Bacterial Strains	Tobramycin (TM)	TM/DEHP	TM/DEHT	TM/DEP	TM/TXIB	TM/DBP	Ceftazidime CAZ	CAZ/DEHP	CAZ/DEHT	CAZ/DEP	CAZ/TXIB	CAZ/DBP
P40	1	2	2	2	2	2	1	1–2	2–4	2–4	2–4	2–4
P41	2	2	2	2	2	2	2	2	2	2	2	2
P42	16	16	16	16	16	16	>16	16	>16	>16	>16	>16
P43	>16	>16	>16	>16	>16	>16	>16	>16	>16	>16	>16	>16
P44	2–4	2	2–4	2–4	2–4	2–4	2	2–4	2–4	2–4	2–4	2–4
P45	2	2	2	2	2	2	4	2–4	2–4	2–4	2–4	2–4
P46	1	1	1	1	1	1	1	1	1	1	1	1
P47	0.5–1	1	1	1	1	1	1	1	1	1	1	1
P48	1	1	1	1	1	1	1	1	1	1	1	1
P49	1	1	1	1	1	1	1	1	1	1	1	1
P50	2	2	2	2	2	2	2	2	2–4	2–4	2–4	2–4
P51	2	2	2–4	2	2	2	2	2	2–4	2–4	2–4	2–4
P52	1	1	1	1	1	1	1	0.5–1	1	1	0.5–1	0.5–1
P53	1	1	1	1	1	1	1	0.5–1	1	1	0.5–1	1
P54	1	1	1	1	1	1	1	0.5–1	0.5–1	1	0.5–1	0.5–1
P55	2	2	2–4	2	2	2	2	2	2–4	2	4	2–4
P56	1	1	1	1	1	1	1	1	1	1	1	1
P57	1	1	1	1	1	1	1	1	1	1	1	1
P58	2	2	2	2	2	2	2	2	2–4	2–4	2–4	2–4
P59	>16	>16	>16	>16	>16	>16	>16	>16	>16	>16	>16	>16
P60	8	8	8	8	8	8	>16	>16	>16	>16	>16	>16
P61	2	2	2	2	2	2	2	2	2	2	2	2
P66	1	1	1	1	1	1	1	1	1–2	2–4	2–4	2–4
P67	0.5	0.5	0.5	0.5	0.5	1	0.5	0.5	0.5	0.5	0.5	0.5
P68	0.5	0.5	0.5	0.5	0.5	1	0.5	0.5	0.5	0.5	0.5	0.5
ATCC27 853	0.5	0.5	0.5	0.5	0.5	0.5	0.5	1	1	1	1	1
